# Establishment of a nomogram based on Lasso Cox regression for albumin combined with systemic immune-inflammation index score to predict prognosis in advanced pancreatic carcinoma

**DOI:** 10.3389/fonc.2025.1447055

**Published:** 2025-04-08

**Authors:** Min Xu, Yu Long, Peisheng Chen, Ang Li, Jian Xin, Yonghua Xu

**Affiliations:** ^1^ The Yancheng Clinical College of Xuzhou Medical University, Yancheng, China; ^2^ Department of General Surgery, The Affiliated Yancheng First Hospital of Nanjing University Medical School, Yancheng, China; ^3^ Department of Clinical Laboratory, The Affiliated Yancheng First Hospital of Nanjing University Medical School, Yancheng, China

**Keywords:** advanced pancreatic carcinoma, LASSO Cox regression, A-SII score, nomogram, overall survival

## Abstract

**Purpose:**

The study aims to establish a nomogram to predict advanced pancreatic carcinoma patients’ overall survival (OS), incorporating albumin combined with systemic immune-inflammation index (A-SII) score and clinical characteristics.

**Methods:**

A retrospective study analyzed the clinical data of 205 advanced pancreatic carcinoma patients without antitumor treatment from the Yancheng No.1 People’s Hospital between October 2011 and June 2023, and the study divided patients into the training set and the validation set randomly at the proportion of three to one. The A-SII score was divided into scores of 0, 1, and 2 according to the different levels of albumin and SII. Receiver operating characteristic (ROC) curves and time-dependent area under the curve were used to evaluate the predictive ability of the A-SII score. The nomogram1 and nomogram2 were established by the multivariate Cox regression and Lasso Cox regression respectively. The study evaluated the discriminability of nomogram1 and nomogram2 based on C-index and ROC curves to obtain the optimal model. Subsequently, we plotted decision curve analyses (DCA) and calibration curves to estimate the clinical benefit and accuracy of nomogram2.

**Results:**

Lasso Cox regression showed that A-SII score, number of organ metastases, tumor size, chemotherapy, targeted therapy, Neutrophil-to-albumin ratio, and lactate dehydrogenase were independent prognostic factors for the OS of advanced pancreatic carcinoma patients. The C-index and ROC curve of the nomogram2 are better than the nomogram1. Subsequently, the DCA and calibration curve of the nomogram2 demonstrate excellent performance.

**Conclusion:**

The nomogram based on the A-SII score and other independent prognostic factors determined by Lasso Cox regression can accurately predict the OS of patients suffering from advanced pancreatic carcinoma.

## 1 Introduction

Pancreatic carcinoma (PC) is an extremely aggressive and fatal malignancy with rapidly rising morbidity and mortality. It ranks as the third cause of cancer-related mortality globally, and its five-year overall survival (OS) probability is just 10% (1–4). In order to improve the OS of patients with advanced pancreatic carcinoma, Multi-disciplinary Treatment and Holistic Integrated Medicine have gradually emerged (5, 6), which aim for early discovery, early diagnosis, and early treatment of PC. However, due to the non-specific symptoms of the early stages of pancreatic carcinoma, most patients were already diagnosed with locally advanced or metastatic pancreatic carcinoma when they were detected, and the overall therapeutic effect of advanced pancreatic carcinoma is not obvious (7, 8). Recently, in addition to chemotherapy, other adjuvant treatments such as targeted therapy and immunotherapy have been gradually applied for advanced pancreatic carcinoma (9, 10). However, the prognoses of different patients with the same therapy methods are quite different, which makes the clinical evaluation of prognosis face challenges (11, 12). Therefore, the study aims to find simple and individualized biomarkers that effectively evaluate patients’ prognoses and guide clinical decisions.

At present, the tumor–node–metastasis (TNM) classification is identified as the optimal staging system for PC. However, patients with the same substage of advanced pancreatic carcinoma show different prognoses due to their heterogeneity (13). In clinical diagnosis and treatment, clinicians commonly rely on tumor imaging features to assess patient prognosis. For patients with advanced pancreatic carcinoma who are unable to undergo surgery, the inability to evaluate regional lymph node metastases makes specific N stages difficult to be determined accurately (14, 15). Therefore, it is urgent to find new biological markers to predict the survival probability of advanced pancreatic carcinoma. Recently, studies have found that some serum markers reflecting the body’s immune inflammation and nutritional status can predict the prognosis of PC, such as neutrophil-to-lymphocyte ratio (NLR), systemic immune-inflammation index (SII), albumin and prognostic nutrition index (PNI), and so on (16–18). However, these markers are single and can’t comprehensively predict the prognosis of PC. In addition, there are rarely studies that investigated the relationship between albumin combined with SII (A-SII) score and prognosis of advanced pancreatic carcinoma.

Therefore, the study aims to explore whether the A-SII score and clinical characteristics could accurately predict the survival probability of advanced pancreatic carcinoma. Meanwhile, to eliminate the multicollinearity between different indicators (19), a nomogram will be constructed based on Lasso Cox regression to guide clinical decisions.

## 2 Materials and methods

### 2.1 Patients selected

The study included 205 advanced pancreatic carcinoma patients without antitumor treatment admitted to the Affiliated Yancheng No.1 People’s Hospital of Nanjing University between October 2011 and June 2023. In this study, advanced pancreatic carcinoma was diagnosed according to the diagnostic criteria of NCCN Clinical Practice Guidelines in Oncology. Inclusion criteria (1): pancreatic carcinoma was diagnosed by cytological biopsy and locally advanced pancreatic carcinoma was diagnosed according to clinical imaging data, or metastatic pancreatic carcinoma (mPC) was confirmed by pathology, (2) age >18 years old, (3) no previous antitumor treatment. Exclusion criteria: (1) patients with other malignant tumors(n=4); (2) patients with incomplete clinical data(n=12); (3) patients lost to follow-up(n=18). According to the above criteria, a number of 205 patients were enrolled in our research (**Supplementary Figure S1**). Since this study was retrospective and just analyzed the clinical data of the included patients and no human specimens were involved, the informed consent form was waived. This study has been approved by the Ethics Committee of the Affiliated Yancheng No.1 People’s Hospital of Nanjing University (approval number: 2024-K-001).

### 2.2 Data elements

The study collected general information and clinical features of the enrolled patients through querying the Hospital Information System. Objective data, including medical records, imaging findings, and laboratory test results, were used as data sources to reduce the influence of subjective factors. Moreover, the data were collected by two designated researchers following strict inclusion and exclusion criteria within the same time period to reduce information bias. General information: age, sex, diabetes; Clinical features: (1) tumor information: liver metastases and other metastases, number of organ metastases, primary site and tumor size; (2) Treatment: radiation, chemotherapy, immunotherapy, targeted therapy; (3) Serological indicators: neutrophil count, lymphocyte count, platelet count, serum albumin, lactate dehydrogenase (LDH), carbohydrate antigen 19-9 (CA19-9). All serological indicators were obtained from venous blood collected with an empty belly within 7 days before the first diagnosis of locally advanced or metastatic pancreatic carcinoma.

### 2.3 Definitions of neutrophil-to-albumin ratio, systemic immune-inflammation index, and A-SII score

Neutrophil-to-albumin ratio (NAR) and SII were calculated according to the following formula: NAR = neutrophil count(×10^9^/L)/albumin (g/L), SII = platelet count (×10^9^/L) × neutrophil count (×10^9^/L)/lymphocyte count (×10^9^/L). In this study, albumin and SII were combined to establish the A-SII score. The optimal cutoff values for albumin and SII were identified through X-tile. According to the best cutoff values of albumin and SII, the A-SII score was divided into three groups, and the specific scoring rules were as follows: the A-SII score of 0 (high albumin and low SII); The A-SII score of 1 (high albumin and high SII or low albumin and low SII); The A-SII score of 2 (low albumin and high SII). Additionally, we evaluated the predictive ability of the A-SII score using receiver operating characteristic (ROC) curves and time-dependent area under the curve (t-AUC) analyses.

### 2.4 Follow-up

We used telephone, text messages, or outpatient reviews to follow up with all enrolled patients, and all patients were followed up until death or December 31, 2023. OS was defined as the time from diagnosis of advanced pancreatic carcinoma to death or the time from diagnosis of advanced pancreatic carcinoma to the end of follow-up. The enrolled patients’ median follow-up time was 677 days (468-886 days).

### 2.5 Statistical analysis

In our study, we used the median as the cutoff value of age, and the optimal cutoff values of other continuous variables were calculated by the X-tile software, which fully considered both the survival time and survival status of patients. Data were analyzed with the use of IBM SPSS Statistics (27.0.1) and R software (4.3.3). The included patients were divided into the training set (n=154) and the validation set (n=51) at random according to the proportion of three to one by R software, each variable between the training set and the validation set had no statistical difference (p > 0.05). A nomogram was developed using the training set, and its predictive performance was validated using the validation set data to reduce selection bias. The study used the chi-square test or Fisher exact test to evaluate the relationship between the A-SII score and clinical data.

The Univariate and multivariate Cox regression were used to confirm the prognostic factors of advanced pancreatic carcinoma. P <0.05 was considered a statistical difference. We used Lasso regression to eliminate the influence of multicollinearity among the factors with P <0.05 based on the univariate Cox regression. R software was used to establish nomogram1 based on the multivariate Cox regression and nomogram2 based on the Lasso Cox regression. In order to confirm the better nomogram, the study used C-index and AUC to compare the discrimination of the two nomograms. The decision curve analyses (DCA) and calibration curve were used to evaluate the clinical benefit and accuracy of the better nomogram. Finally, the enrolled patients were divided into low risk group and high risk group by the median of total points calculated through the better nomogram. The Kaplan-Meier (K-M) survival difference analysis was performed by the log-rank test. In this study, we considered that P<0.05 has a statistical difference.

## 3 Results

### 3.1 Patient characteristics

A number of 205 patients with advanced pancreatic carcinoma without antitumor therapy were admitted to our study, and all patients were divided into the training set (n=154) and validation set (n=51) at random according to the proportion of three to one. The median age of the study population was 67 years (38-95 years). This study contained 133 males (64.9%) and 72 females (35.1%). In addition, 77 patients (37.6%) had diabetes history in the study. Otherwise, the majority of patients had liver metastases (69.3%). In terms of adjuvant therapy, the largest number of patients were chemotherapy (58.5%). The rest patients did not receive chemotherapy, potentially due to factors such as advanced age, weakened physical condition, severe cancer pain, or low willingness to undergo treatment. More clinical features and treatment regimens of patients can be found in **Table 1**.

**Table 1:** Comparison of clinical data between the training set and validation set.

Characteristics	Total (n=205)	Training	Validation	P value
		set (n=154)	set (n=51)	
Age, *n* (*%*)				0.906
≤67 years	112 (54.6)	85 (55.2)	27 (52.9)	
>67 years	93 (45.4)	69 (44.8)	24 (47.1)	
Sex, *n* (*%*)				1
Male	133 (64.9)	100 (64.9)	33 (64.7)	
Female	72 (35.1)	54 (35.1)	18 (35.3)	
Diabetes, *n* (*%*)				1
No	128 (62.4)	96 (62.3)	32 (62.7)	
Yes	77 (37.6)	58 (37.7)	19 (37.3)	
Liver metastases, *n* (*%*)				0.681
No	63 (30.7)	49 (31.8)	14 (27.5)	
Yes	142 (69.3)	105 (68.2)	37 (72.5)	
Other metastases, *n* (*%*)				0.469
No	130 (63.4)	95 (61.7)	35 (68.6)	
Yes	75 (26.6)	59 (38.3)	16 (31.4)	
Number of organ metastases, *n* (*%*)				0.227
0	27 (13.2)	22 (14.3)	5 (9.8)	
1	128 (62.4)	91 (59.1)	37 (72.5)	
≥2	50 (24.4)	41 (26.6)	9 (17.6)	
Primary site, *n* (*%*)				0.05
Head of pancreas	63 (30.7)	42 (27.3)	21 (41.2)	
Neck of pancreas	8 (3.9)	7 (4.5)	1 (2.0)	
Body of pancreas	33 (16.1)	27 (17.5)	6 (11.8)	
Tail of pancreas	44 (21.5)	29 (18.8)	15 (29.4)	
Overlapping lesion of pancreas	57 (27.8)	49 (31.8)	8 (15.7)	
Tumor size, *n* (*%*)				0.594
≤52 mm	153 (74.6)	113 (73.4)	40 (78.4)	
>52 mm	52 (25.4)	41 (26.6)	11 (21.6)	
Chemotherapy, *n* (*%*)				0.832
No	85 (41.5)	65 (42.2)	20 (39.2)	
Yes	120 (58.5)	89 (57.8)	31 (60.8)	
Radiation, *n* (*%*)				0.69
No	182 (88.8)	138 (89.6)	44 (86.3)	
Yes	23 (11.2)	16 (10.4)	7 (13.7)	
Immunotherapy, *n* (*%*)				0.802
No	177 (86.3)	134 (87.0)	43 (84.3)	
Yes	28 (13.7)	20 (13.0)	8 (15.7)	
Targeted therapy, *n* (*%*)				0.917
No	178 (86.8)	133 (86.4)	45 (88.2)	
Yes	27 (13.2)	21 (13.6)	6 (11.8)	
LDH, *n* (*%*)				0.18
≤264 U/L	142 (69.3)	111 (72.1)	31 (60.8)	
>264 U/L	63 (30.7)	43 (27.9)	20 (39.2)	
CA19-9, *n* (*%*)				0.255
≤10.5 U/mL	23 (11.2)	20 (13.0)	3 (5.9)	
>10.5 U/mL	182 (88.8)	134 (87.0)	48 (94.1)	
NAR, *n* (*%*)				0.708
≤0.10	87 (42.4)	67 (43.5)	20 (39.2)	
>0.10	118 (57.6)	87 (56.5)	31 (60.8)	
A-SII score, *n* (*%*)				0.152
0	95 (46.3)	74 (48.1)	21 (41.2)	
1	69 (33.7)	54 (35.1)	15 (29.4)	
2	41 (20.0)	26 (16.9)	15 (29.4)	

### 3.2 A-SII score establishment, assessment, and relationship to clinical data

As shown in **Supplementary Figure S2**, the optimal cutoff value determined by the X-tile software for albumin was 36.8(g/L), and for SII was 930.9(×10^9^/L). Univariate and multivariate Cox regression analyses identified albumin and SII as independent prognostic factors in advanced pancreatic carcinoma (P < 0.05, **Supplementary Table S1**). Therefore, we combined albumin and SII to establish the A-SII score. The 12-month ROC curves of the A-SII score indicated that its AUC was 0.741, which was superior to 0.684 for albumin and 0.651 for SII (**Supplementary Figure S3A**). Furthermore, the t-AUC curves indicated that the A-SII score exhibited superior predictive performance compared to albumin or SII individually (**Supplementary Figure S3B**).

As shown in **Table 2**, there were 95 patients (46.3%), 69 patients (33.7%), and 41 patients (20.0%) with A-SII scores of 0, 1, and 2 respectively. In addition, the A-SII score was significantly relevant with age (P=0.048), primary site (P=0.024), tumor size (P=0.026), LDH(P=0.001), and NAR (P < 0.001). However, the A-SII score had no significant correlations with sex, diabetes, and other markers (P > 0.05).

**Table 2:** Relationships between A-SII score and clinical data (n=205).

Factors	A-SII score			X²	P value
	0(n=95)	1(n=69)	2(n=41)		
Age, *n*				6.08	0.048
≤67 years	60	35	17		
>67 years	35	34	24		
Sex, *n*				4.801	0.091
Male	68	38	27		
Female	27	31	14		
Diabetes, *n*				1.027	0.599
No	62	43	23		
Yes	33	26	18		
Liver metastases, *n*				0.368	0.832
No	30	22	11		
Yes	65	47	30		
Other metastases, *n*				2.165	0.339
No	65	42	23		
Yes	30	27	18		
Number of organ metastases, *n*				5.787	0.216
0	13	11	3		
1	65	38	25		
≥2	17	20	13		
Primary site, *n*				17.593	0.024
Head of pancreas	28	23	12		
Neck of pancreas	5	3	0		
Body of pancreas	17	9	7		
Tail of pancreas	17	10	17		
Overlapping lesion of pancreas	28	24	5		
Tumor size, *n*				7.308	0.026
≤52 mm	79	48	26		
>52 mm	16	21	15		
Chemotherapy, *n*				3.781	0.151
No	34	29	22		
Yes	61	40	19		
Radiation, *n*				2.41	0.3
No	84	59	39		
Yes	11	10	2		
Immunotherapy, *n*				5.902	0.052
No	77	65	35		
Yes	18	4	6		
Targeted therapy, *n*				2.09	0.352
No	79	62	37		
Yes	16	7	4		
LDH, *n*				13.444	0.001
≤264 U/L	76	46	20		
>264 U/L	19	23	21		
CA19-9, *n*				0.606	0.739
≤10.5 U/mL	10	7	6		
>10.5 U/mL	85	62	35		
NAR, *n*				42.204	<0.001
≤0.10	59	27	1		
>0.10	36	42	40		

Statistical differences are indicated in bold.

### 3.3 Independent prognostic factors for advanced pancreatic carcinoma

As shown in **Table 3**, 16 variables were subjected to univariate Cox regression, and the results showed that age, other metastases, number of organ metastases, tumor size, chemotherapy, targeted therapy, LDH, NAR, A-SII score had significant correlation with the prognosis of the patients with advanced pancreatic carcinoma (P <0.05). Then, variables with statistical differences in the univariate Cox regression were admitted into the multivariate Cox regression. The final results showed that the number of organ metastases, tumor size, chemotherapy, targeted therapy, NAR, and A-SII score were independent prognostic factors for OS.

**Table 3:** Univariate and multivariate Cox regression analyses of all variables for overall survival of advanced pancreatic carcinoma patients.

Variables	Univariate analysis		Multivariate analysis	
	Hazard Ratio(95%CI)	P value	Hazard Ratio(95%CI)	P value
Age				
≤67 years	Reference			
>67 years	1.442 (1.020-2.038)	**0.038**	0.892 (0.600-1.325)	0.571
Sex				
Male	Reference			
Female	1.051 (0.732-1.509)	0.788		
Diabetes				
No	Reference			
Yes	0.900 (0.629-1.286)	0.563		
Liver metastases				
No	Reference			
Yes	1.274 (0.874-1.856)	0.208		
Other metastases				
No	Reference			
Yes	1.541 (1.085-2.187)	**0.016**	0.673 (0.374-1.212)	0.187
Number of organ metastases				
0	Reference			
1	0.817 (0.574-1.164)	0.263	1.382 (0.745-2.565)	0.305
≥2	1.921 (1.310-2.817)	**<0.001**	2.499 (1.027-6.080)	**0.043**
Primary site				
Head of pancreas	Reference			
Neck of pancreas	0.920 (0.375-2.257)	0.855		
Body of pancreas	0.922 (0.591-1.438)	0.721		
Tail of pancreas	1.103 (0.721-1.687)	0.653		
Overlapping lesion of pancreas	1.345 (0.930-1.946)	0.116		
Tumor size				
≤52 mm	Reference			
>52 mm	1.656 (1.133-2.418)	**0.009**	1.584 (1.068-2.351)	**0.022**
Chemotherapy				
No	Reference			
Yes	0.542 (0.383-0.766)	**<0.001**	0.569 (0.384-0.843)	**0.005**
Radiation				
No	Reference			
Yes	0.730 (0.393-1.356)	0.319		
Immunotherapy				
No	Reference			
Yes	0.725 (0.434-1.208)	0.217		
Targeted therapy				
No	Reference			
Yes	0.457 (0.261-0.799)	**0.006**	0.478 (0.267-0.855)	**0.013**
LDH				
≤264 U/L	Reference			
>264 U/L	2.080 (1.435-3.016)	**<0.001**	1.359 (0.910-2.031)	0.134
CA19-9				
≤10.5 U/mL	Reference			
>10.5 U/mL	0.704 (0.426-1.163)	0.17		
NAR				
≤0.10	Reference			
>0.10	2.511 (1.741-3.622)	**<0.001**	1.676 (1.080-2.602)	**0.021**
A-SII score				
0	Reference			
1	1.650 (1.151-2.365)	**0.006**	2.005 (1.298-3.099)	**0.002**
2	3.211 (2.038-5.059)	**<0.001**	3.308 (1.872-5.847)	**<0.001**

Statistical differences are indicated in bold.

**Figure 1:**
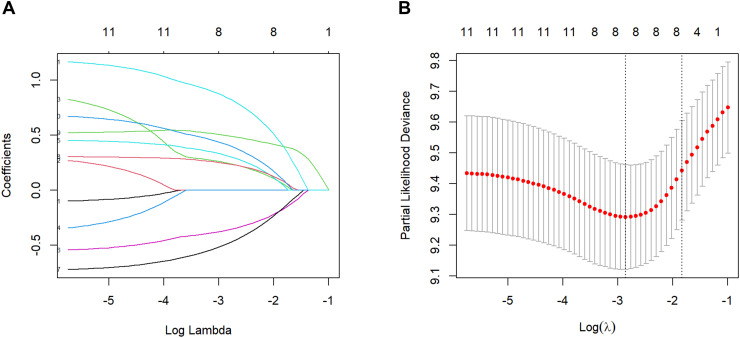
Factors selection by the Lasso regression. **(A)** Lasso coefficient profile of the 11 factors. **(B)** 7 prognostic factors were selected based on 1 standard error criteria of the minimum considered as the optimal parameter (lambda) in the Lasso model.

Meanwhile, considering the possible collinearity relationship between the variables, we used Lasso regression to analyze the variables with statistical differences in the univariate Cox regression, and the variation characteristics of the coefficients of each variable are shown in **Figure 1A**. By the cross-validation method, 7 variables were selected at one standard error criteria of minimum which was the optimal penalty coefficient, including the number of organ metastases, tumor size, chemotherapy, targeted therapy, NAR, LDH, and A-SII score confirmed as the independent prognostic factors for OS (**Figure 1B**).

### 3.4 Nomogram establishment and validation

**Figure 2:**
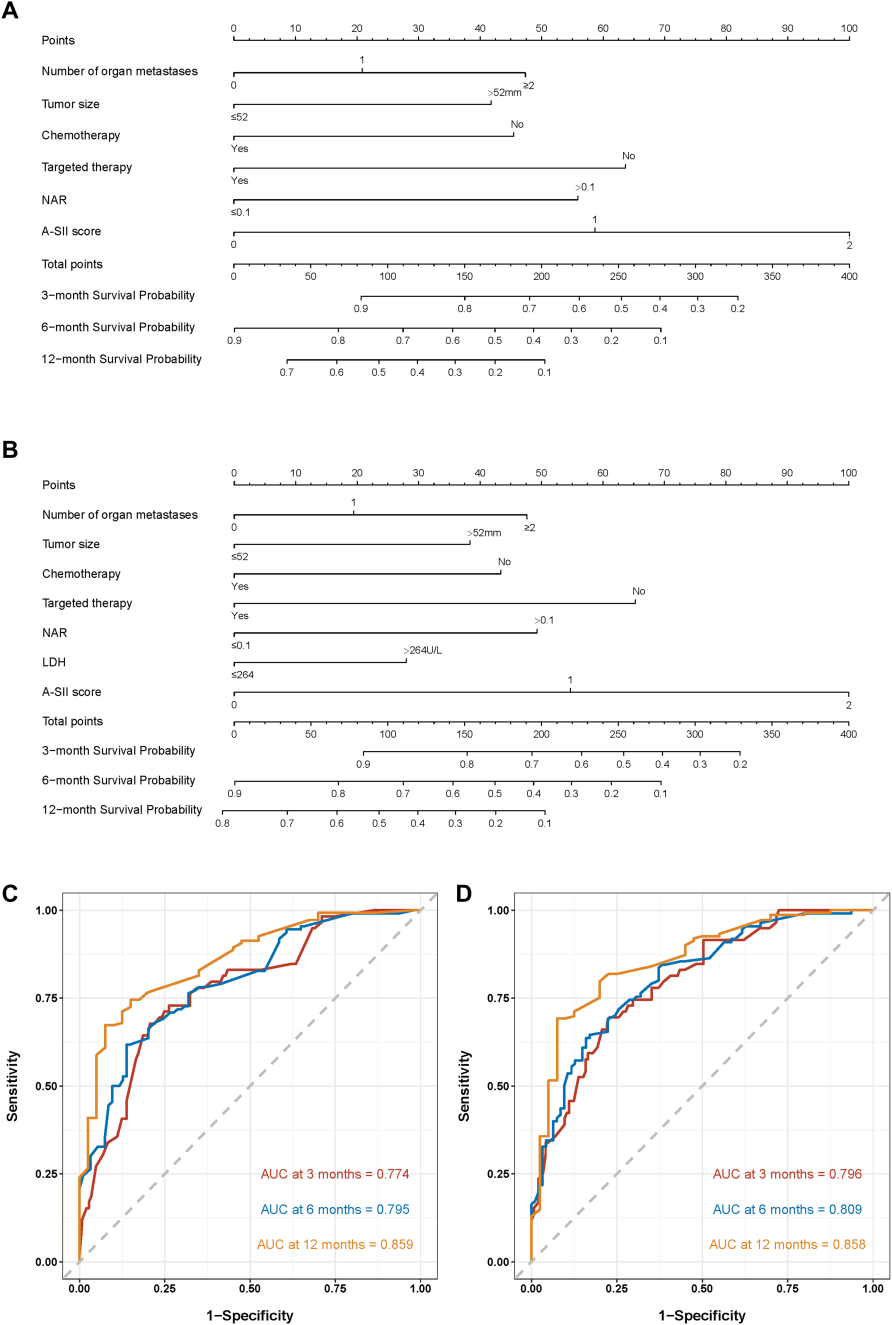
Nomograms to predict the probabilities of 3-, 6-, and 12-month OS and their ROC curves. **(A)** The nomogram1 based on the univariate and multivariate Cox regression; **(B)** The nomogram2 on the basis of the Lasso Cox regression. **(C)** ROC curves of nomogram1; **(D)** ROC curves of nomogram2.

Firstly, we established nomogram1 to predict the 3-, 6-, and 12-month OS based on the univariate and multivariate Cox regression (**Figure 2A**), and meanwhile, nomogram2 was constructed to predict the 3-, 6-, and 12-month OS based on the Lasso Cox regression (**Figure 2B**). The C-index of the multivariate Cox regression was 0.728(95%CI: 0.674-0.763) in the training set and 0.779(95%CI: 0.684-0.822) in the validation set. The C-index of the Lasso Cox regression was 0.735(95%CI: 0.673-0.767) in the training set and 0.791(95%CI: 0.719-0.831) in the validation set. It is clear that the C-index of nomogram2 is superior to nomoram1. Then, the ROC curves of the two nomograms were plotted, and the AUC of nomogram1 in all enrolled patients for predicting 3-, 6-, and 12-month OS respectively reached 0.774,0.795,0.859 (**Figure 2C**). ROC analysis of nomogram2 in all enrolled patients showed that AUC of 3-, 6-, and 12-month OS respectively reached 0.796, 0.809, 0.858 (**Figure 2D**). Considering the C-index and ROC curves in an integrated manner, nomogram2 was superior to nomogram1, so we selected nomogram2 as our visualization model to predict 3-, 6-and 12-month OS.

**Figure 3:**
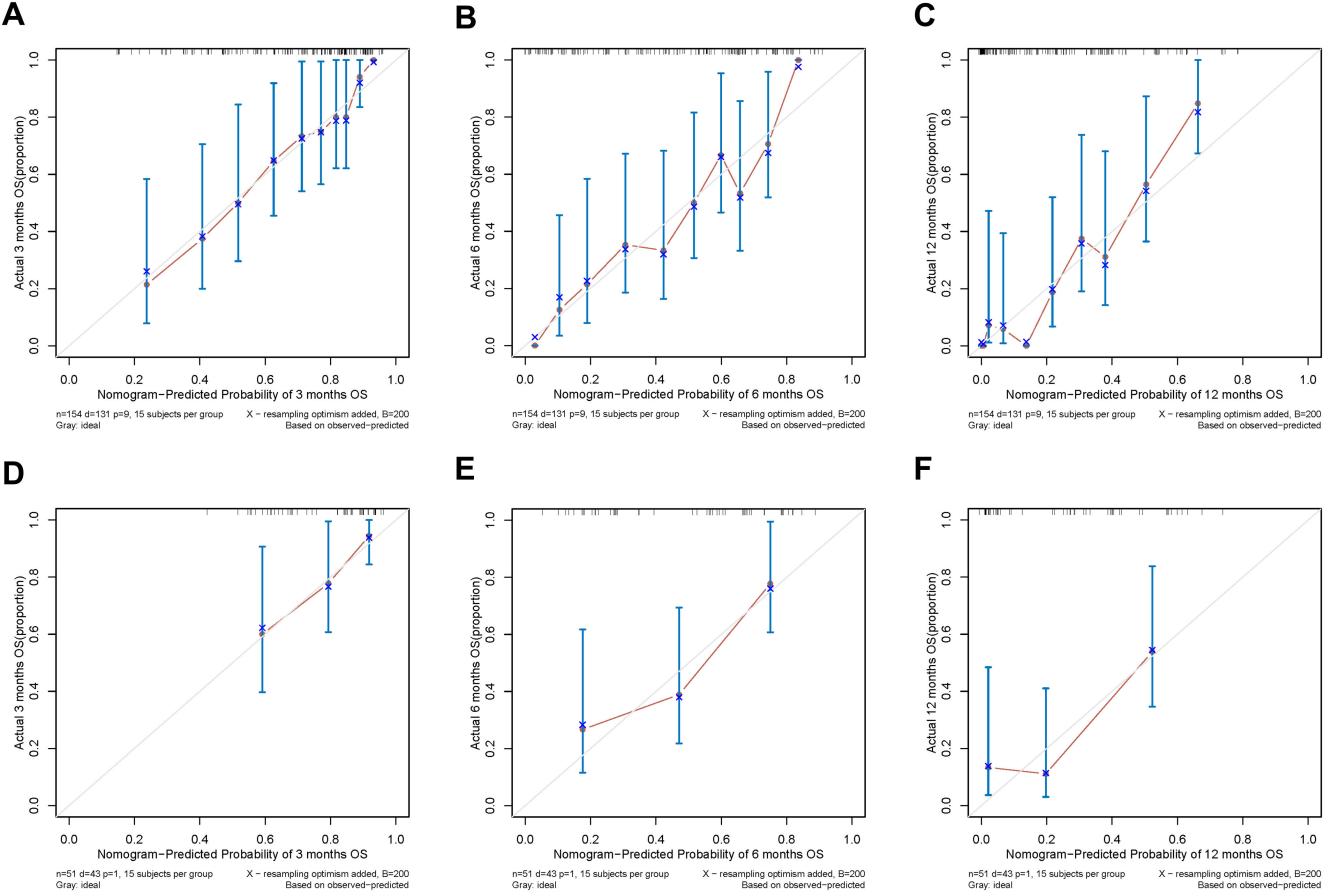
Calibration curves of the nomogram2. **(A-C)** the training set; **(D-F)** the validation set.

**Figure 4:**
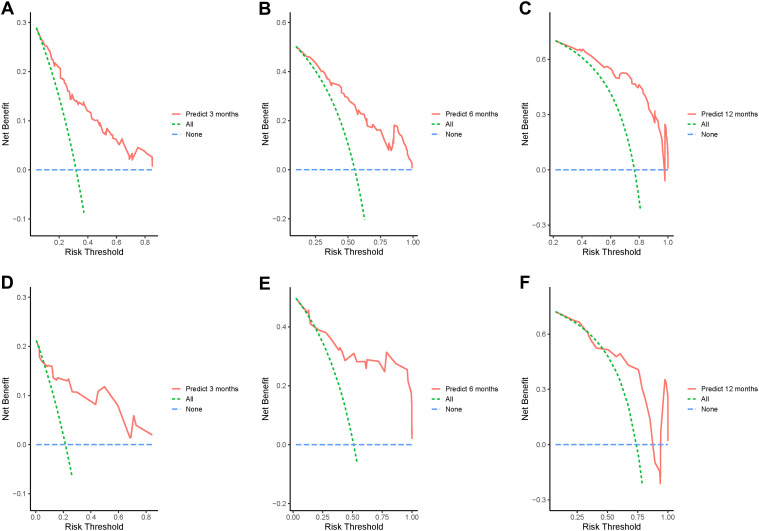
Decision curve analysis of the nomogram2. **(A-C)** the training set; **(D-F)** the validation set.

In addition, the calibration curves of nomogram2 indicated satisfied consistency between actual observation and prediction (**Figure 3**). To quantify the utility of nomogram2 at specific clinical decision thresholds, DCA curves were plotted, and the result showed that nomogram2 had favorable net clinical benefit (**Figure 4**).

### 3.5 Survival analysis

**Figure 5:**
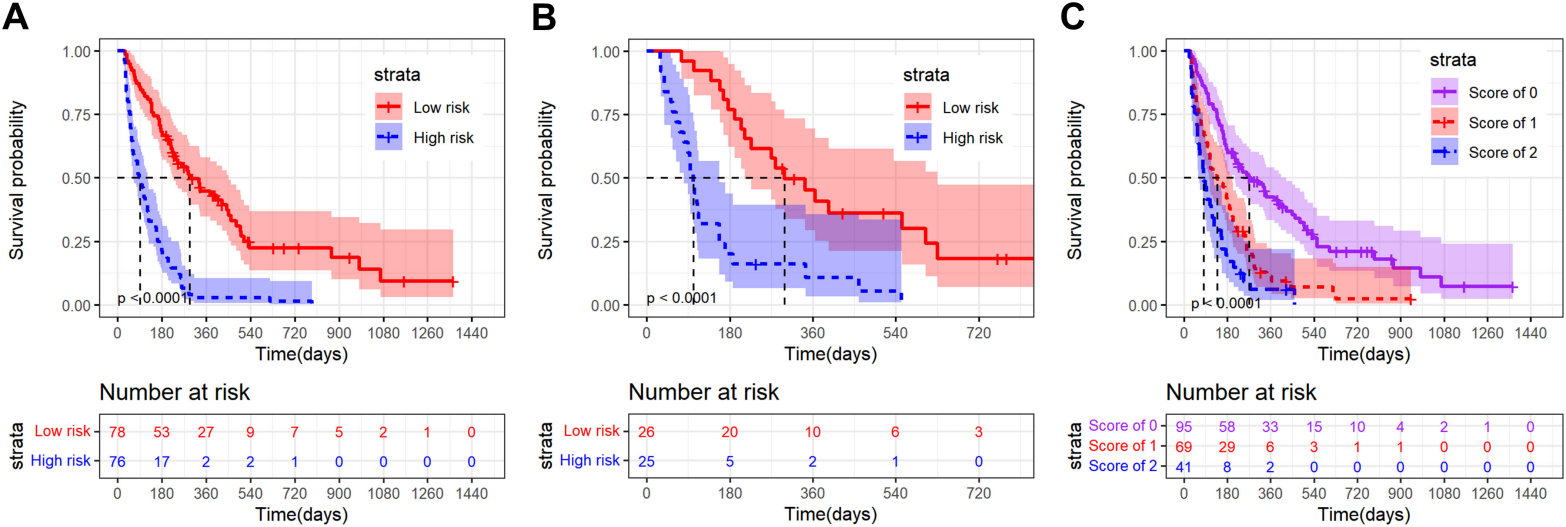
Kaplan-Meier survival curves of advanced pancreatic carcinoma patients divided into different strata according to the nomogram2 total points or A-SII score. **(A)** K-M curve of the training set in low risk and high risk groups on the basis of the nomogram2 total points; **(B)** K-M curve of the validation set in low risk and high risk groups on the basis of the nomogram2 total points; **(C)** K-M curve of all patients in the A-SII score of 0,1 and 2 groups.

The nomogram2 total points were divided into low risk group and high risk group by the median, and then the K-M survival curves of the training set and validation set revealed a significant difference in survival probability between the two groups (p < 0.0001, **Figures 5A, B**). In addition, as shown in **Figure 2B**, the A-SII score was the most important factor of all those independent prognostic factors, and we plotted K-M curves of A-SII scores of 0 (n=95), 1 (n=69), and 2 (n=41). The three groups had significant differences in survival probability (p < 0.0001), and patients with low scores had better prognoses than those with high scores (**Figure 5C**). Additionally, a total of 178 patients with mPC were analyzed as a subgroup using K-M survival analysis. In the mPC subgroup, the low risk group had significantly better survival probability than the high risk group, as determined by total points from nomogram2 (p < 0.0001, **Supplementary Figures S4A, B**). K-M curves for patients with mPC, categorized by A-SII scores of 0 (n=82), 1 (n=58), and 2 (n=38), showed significant differences in survival probabilities among the three groups (p < 0.0001, **Supplementary Figure S4C**). Patients with high A-SII scores have significantly worse prognoses than those with low A-SII scores in mPC.

## 4 Discussion

Since pancreatic carcinoma is hardly diagnosed at the early stages and easily occurs metastases, patients often have progressed to advanced pancreatic carcinoma when diagnosed, and their prognoses are very poor (2, 3). In recent years, immune-inflammatory responses and nutritional status have been found to be relevant to the prognosis of patients with PC, however, the relevant markers of pancreatic carcinoma prognosis are relatively single at present (20, 21). Therefore, in this study, albumin and SII were combined to obtain the A-SII score to make up for the deficiency of a single marker, and a nomogram was constructed on basis of the Lasso Cox regression for predicting the OS of advanced pancreatic carcinoma patients.

Previous studies have shown that the nutritional status and the systemic immune inflammatory response are involved in the occurrence and development of malignant tumors, also influencing the prognoses of patients (17, 18). Serum albumin serves not only as a crucial indicator of nutritional status but also as a significant marker of liver protein synthesis efficiency (22). Previous studies have revealed that tumor cells can produce and release some inflammatory mediators, such as tumor necrosis factor-alpha and interleukin-6, which may inhibit the synthesis of albumin in the liver, potentially resulting in hypoproteinemia (23, 24). Mitsunaga et al. (25)reported that patients with advanced pancreatic carcinoma who exhibited higher levels of IL-6 had lower OS. Additionally, the serum albumin level not only reflects nutritional status but is also associated with the inflammatory response. Cytokines released by inflammatory cells increase microvascular permeability, resulting in greater extravasation of serum albumin through the blood vessel wall (26). Previous studies have demonstrated that serum albumin levels are strongly associated with the prognoses of various malignant tumors, including breast cancer and PC (27). Recently, the ratio of albumin to serum indices, such as C-reactive protein (28) and fibrinogen (29), has been frequently used to predict the prognosis of PC, providing a reference for constructing the A-SII score in this study. Recently, numerous studies have demonstrated that SII is closely associated with the prognoses of patients with advanced pancreatic carcinoma (30, 31). SII is calculated based on neutrophils, platelets and lymphocytes, all of which are involved in cancer progression. Neutrophils are often recruited into tumor tissues to differentiate into tumor-associated neutrophils and contribute to the formation of tumor microenvironment. Moreover, neutrophil extracellular traps (NETs) generated by neutrophils can enhance tumor cell proliferation and facilitate cancer cell invasion and metastasis (32, 33). Platelets not only release various pro-survival, pro-angiogenic, and immunomodulatory factors to establish and sustain the primary and metastatic tumor microenvironment but also shield tumor cells from immune clearance (34). Conversely, lymphocytes primarily inhibit tumor proliferation and migration while inducing tumor cell apoptosis. The above explanation may provide further clarity on the association between SII and the prognosis of patients with advanced pancreatic cancer. We observed that most previous studies focused on the prognostic value of single indicators, whereas relatively few explored the prognosis of advanced pancreatic carcinoma using integrated indicators of nutritional status, immunity, and inflammation. This study is the first to investigate the prognostic value of albumin combined with SII in patients with advanced pancreatic carcinoma. The t-AUC analysis confirmed that the A-SII score outperformed albumin or SII alone in predicting the prognosis of advanced pancreatic carcinoma. Furthermore, we found that the A-SII score, as one of the independent prognostic factors, contributed the largest contribution to the nomogram. This indicates that combining albumin and SII is essential for the prediction model and assists clinicians in more accurately estimating patient survival probabilities.

The clinical data and follow-up data of 205 patients with advanced pancreatic carcinoma who had not received antitumor therapy were retrospectively analyzed. The multivariate Cox regression and Lasso Cox regression were used to confirm the independent prognostic factors. Through the comprehensive comparison of C-index and AUC values, nomogram2 established by the Lasso Cox regression which identified 7 variables, including the number of organ metastases, tumor size, chemotherapy, targeted therapy, NAR, LDH, A-SII score, is superior to nomogram1 based on the multivariate Cox regression. Patients with advanced pancreatic carcinoma often develop metastases, and our study showed that patients with multiple metastases had poorer prognoses, consistent with Feng et al.’s study (35), which indicated that patients with multiple metastases in metastatic pancreatic carcinoma had lower OS. In a study of 1,898 patients with liver metastases in PC (36), Shi et al. identified tumor size as an independent prognostic variable, similar to our findings. Chemotherapy is the first-line treatment for advanced pancreatic carcinoma, and multiple studies have confirmed that chemotherapy improves clinical outcomes in advanced pancreatic carcinoma (36, 37), findings consistent with ours. However, for patients with advanced pancreatic carcinoma, single chemotherapy does not meet the demands of clinical multimodal therapy. Recent studies have shown that targeted therapy has potential for clinical application as a novel anti-tumor strategy, as certain clinical trials targeting aberrant pathways and molecular abnormalities have yielded promising results. Targeted therapy has become a new anti-tumor approach in clinical practice. Erlotinib combined with selumetinib demonstrates antitumor efficacy in locally advanced or metastatic pancreatic ductal adenocarcinoma (38). IGF-1R antagonist (MK-0646) in combination with gemcitabine can synergistically enhance OS. This study demonstrates that targeted therapy can also improve OS among advanced pancreatic carcinoma (39, 40). Currently, limited research has explored the relationship between NAR and the prognosis of advanced pancreatic carcinoma. Tingle et al. reported that NAR combined with CA19-9 could effectively predict OS among patients with palliative pancreatic cancer (41). Our study further validated that NAR is a significant prognostic factor in advanced pancreatic carcinoma. It is well known that tumor cells primarily depend on anaerobic glycolysis for energy production, and LDH, a key enzyme in tumor cell metabolism, plays a crucial role. Our results indicate that elevated LDH levels are linked to poor prognosis in advanced pancreatic carcinoma. Similar findings have been observed in non-small cell lung cancer and colorectal cancer (42, 43).

Although CA19-9 has long been considered to play an important role in the diagnosis and prognosis of PC, this study determined that CA19-9 is not an independent prognostic factor in advanced pancreatic carcinoma. Our analysis may be related to the following reasons (1): 5%-10% of patients lack the Lewis blood group antigens, leading to negative CA19-9 results (44); (2) Obstructive jaundice caused by pancreatic head cancer may lead to elevated CA19-9 levels; (3) Studies have shown that dynamic changes in CA19-9 levels may better facilitate the evaluation of PC prognosis (37). In the phase 3 Metastatic Pancreatic Adenocarcinoma Clinical Trial, CA19-9 levels were not significantly associated with the prognosis of metastatic pancreatic adenocarcinoma in multivariate Cox regression analysis (45). Moreover, in exploring the prognostic value of NLR in mPC, a finding found that CA19-9 levels were not correlated with OS (46), which is consistent with ours. In contrast, some studies have shown that CA19-9 levels are strongly correlated with OS in advanced pancreatic carcinoma (47, 48). Given the contradictory evidence regarding the prognostic value of CA19-9, additional robust clinical studies are required to confirm these findings beyond the factors analyzed in our study.

At present, Lasso Cox regression is widely used in gene screening (49), but recently several studies have shown that Lasso Cox regression has important value in screening clinical indicators and constructing predictive models. Zhou D et al. used Lasso Cox regression to establish a nomogram of alpha-fetoprotein-negative hepatocellular carcinoma patients without surgery (50). Fan X et al. established a nomogram based on Lasso Cox regression for predicting OS of patients with T1b esophageal cancer treated by endoscopy (51). However, Lasso Cox regression is rarely used in PC. In order to eliminate the problem of multicollinearity between variables, this study constructed the nomogram2 on the basis of Lasso Cox regression to predict the 3-, 6-, and 12-month OS of patients with advanced pancreatic carcinoma, which showed better prediction performance than the nomogram1 on the basis of multivariate Cox regression. The calibration curve and DCA curve show that nomogram2 has good accuracy and clinical net benefit.

Although this study proposed for the first time that the A-SII score is a crucial independent prognostic factor for advanced pancreatic carcinoma, it still has its limitations. Firstly, the study was retrospective and restricted by the number of samples, which may cause selection bias. Secondly, it was a single-center study that lacked a corresponding external validation set. We look forward to conducting large sample studies to further confirm the current conclusions in the near future.

## 5 Conclusion

A-SII score is a crucial independent prognostic factor for patients with advanced pancreatic carcinoma. In this study, a nomogram based on Lasso Cox regression was established for predicting advanced pancreatic cancer patients 3-,6- and 12-month OS, which has good predictive ability and could accurately distinguish low risk and high risk groups of advanced pancreatic carcinoma.

## Data Availability

The raw data supporting the conclusions of this article will be made available by the authors, without undue reservation.
