# Supplementary material for: Establishment of a nomogram based on Lasso Cox regression for albumin combined with systemic immune-inflammation index score to predict prognosis in advanced pancreatic carcinoma
Source: Front Oncol. 2025 Apr 8;15:1447055. doi: 10.3389/fonc.2025.1447055 (PMC12011609; doi:10.3389/fonc.2025.1447055)
Supplement: Supplementary file 1 [file DataSheet1.pdf]

Supplementary Material

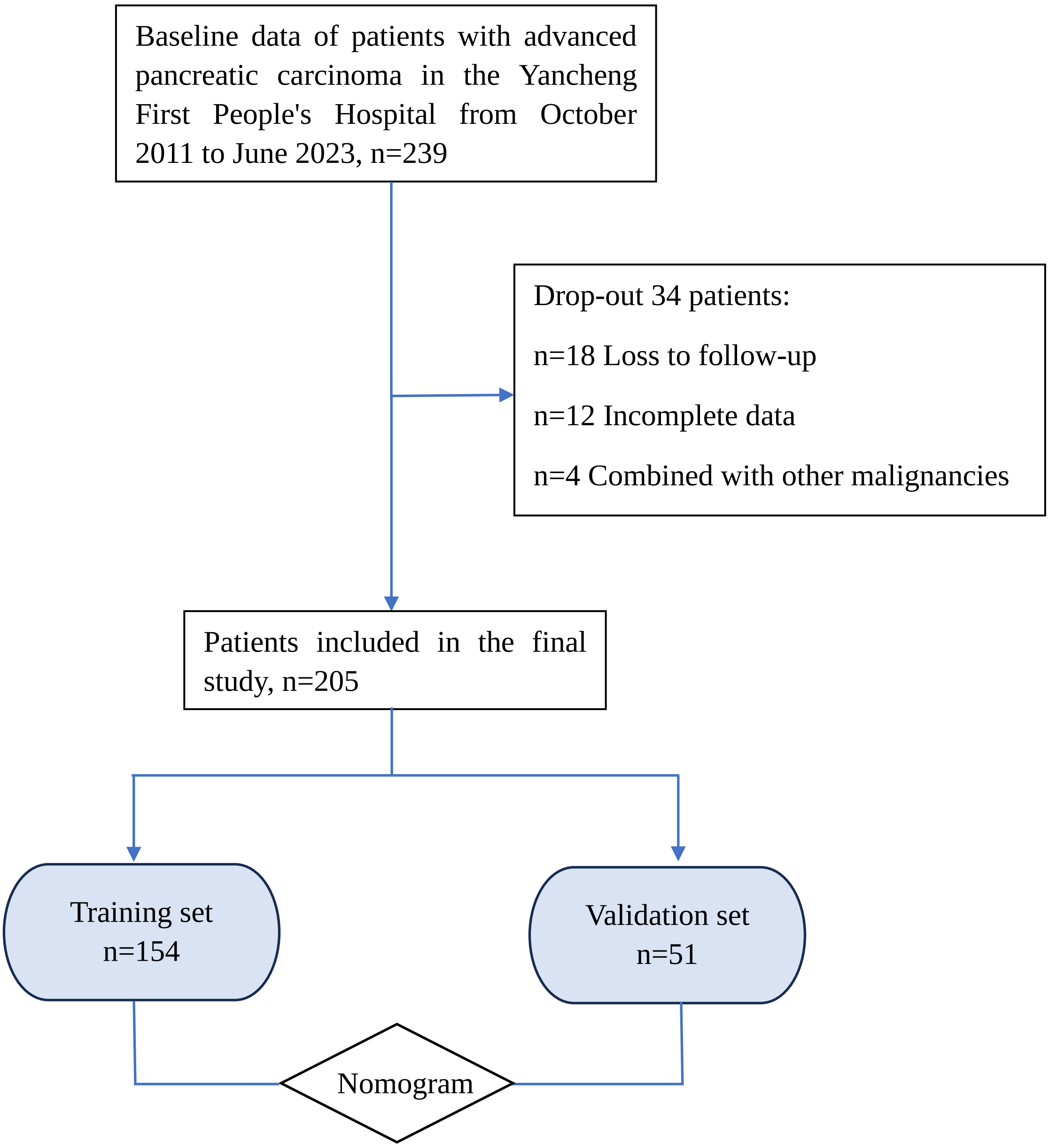

Figure S1. Flowchart illustrating the study design and patients selection process.

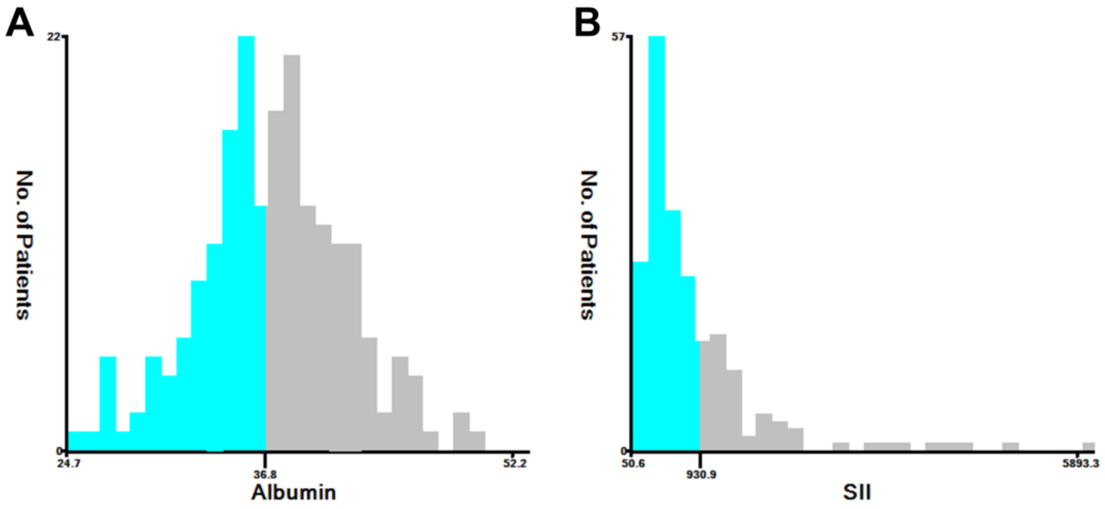

Figure S2. Optimal cutoff values for Albumin and SII determined using X-tile software: (A) Albumin; (B) SII.

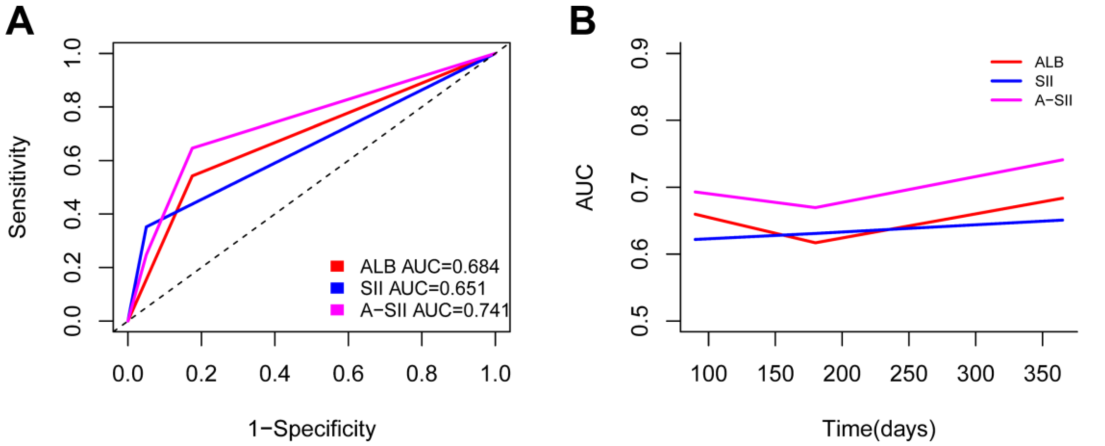

Figure S3. (A) Receiver operating characteristic (ROC) curves for ALB, SII, and A-SII. (B) Time-dependent area under the curve (t-AUC) analysis of ALB, SII, and A-SII. (ALB stands for albumin.)

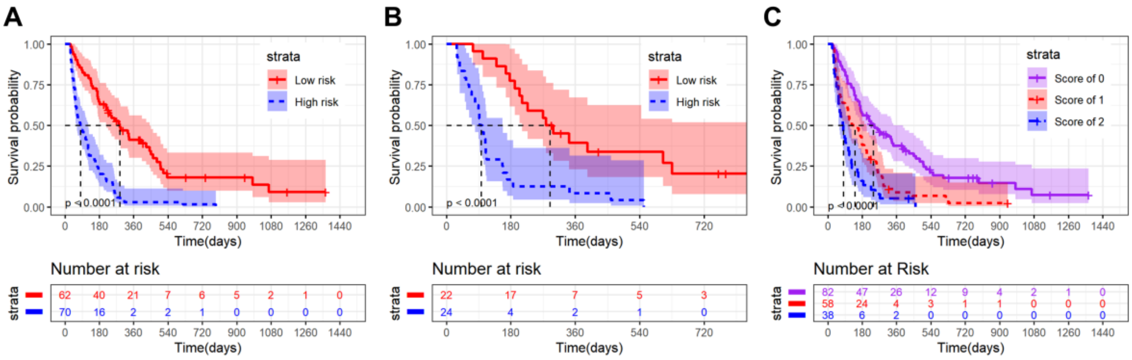

Figure S4. Kaplan-Meier (K-M) survival analysis for metastatic pancreatic carcinoma: (A) Training set, (B) Validation set, (C) A-SII score.

TABLE S1. Univariate and multivariate Cox regression analyses of Albumin, SII and other variables for overall survival of advanced pancreatic carcinoma patients.

Variables	Univariate analysis		Multivariate analysis	
	Hazard Ratio(95%CI)	P value	Hazard Ratio(95%CI)	P value
Age				
≤67 years	Reference			
>67 years	1.442 (1.020-2.038)	**0.038**	0.899 (0.601-1.345)	0.604
Sex				
Male	Reference			
Female	1.051 (0.732-1.509)	0.788		
Diabetes				
No	Reference			
Yes	0.900 (0.629-1.286)	0.563		
Liver metastases				
No	Reference			
Yes	1.274 (0.874-1.856)	0.208		
Other metastases				
No	Reference			
Yes	1.541 (1.085-2.187)	**0.016**	0.684 (0.381-1.226)	0.202
Number of organ metastases				
0	Reference			
1	0.817 (0.574-1.164)	0.263	1.378 (0.743-2.557)	0.309
≥2	1.921 (1.310-2.817)	**<0.001**	2.478 (1.020-6.023)	**0.045**
Primary site				
Head of pancreas	Reference			
Neck of pancreas	0.920 (0.375-2.257)	0.855		
Body of pancreas	0.922 (0.591-1.438)	0.721		
Tail of pancreas	1.103 (0.721-1.687)	0.653		
Overlapping lesion of pancreas	1.345 (0.930-1.946)	0.116		
Tumor size				
≤52 mm	Reference			
>52 mm	1.656 (1.133-2.418)	**0.009**	1.595 (1.076-2.366)	**0.020**
Chemotherapy				
No	Reference			
Yes	0.542 (0.383-0.766)	**<0.001**	0.574 (0.388-0.850)	**0.005**
Radiation				
No	Reference			
Yes	0.730 (0.393-1.356)	0.319		
Immunotherapy				
No	Reference			
Yes	0.725 (0.434-1.208)	0.217		

TABLE S1. continued

Targeted therapy				
No	Reference			
Yes	0.457 (0.261-0.799)	**0.006**	0.473 (0.263-0.848)	**0.012**
LDH				
≤264 U/L	Reference			
>264 U/L	2.080 (1.435-3.016)	**<0.001**	1.383 (0.930-2.057)	0.109
CA19-9				
≤10.5 U/mL	Reference			
>10.5 U/mL	0.704 (0.426-1.163)	0.170		
NAR				
≤0.10	Reference			
>0.10	2.511 (1.741-3.622)	**<0.001**	1.655 (1.054-2.601)	**0.029**
Albumin				
≤36.8 g/L	Reference			
>36.8 g/L	0.443 (0.311-0.632)	**<0.001**	0.538 (0.361-0.799)	**0.002**
SII				
≤930.9 $\times 10^9$/L	Reference			
>930.9 $\times 10^9$/L	3.318 (2.196-5.012)	**<0.001**	1.814 (1.120-2.936)	**0.015**
